# MacroD1 Is a Promiscuous ADP-Ribosyl Hydrolase Localized to Mitochondria

**DOI:** 10.3389/fmicb.2018.00020

**Published:** 2018-01-23

**Authors:** Thomas Agnew, Deeksha Munnur, Kerryanne Crawford, Luca Palazzo, Andreja Mikoč, Ivan Ahel

**Affiliations:** ^1^Sir William Dunn School of Pathology, University of Oxford, Oxford, United Kingdom; ^2^Division of Molecular Biology, Ruđer Bošković Institute, Zagreb, Croatia

**Keywords:** PARP, DNA damage repair, hydrolase, mitochondria, ADP-ribosylation, ADP-ribose, macrodomain

## Abstract

MacroD1 is a macrodomain containing protein that has mono-ADP-ribose hydrolase enzymatic activity toward several ADP-ribose adducts. Dysregulation of MacroD1 expression has been shown to be associated with the pathogenesis of several forms of cancer. To date, the physiological functions and sub-cellular localization of MacroD1 are unclear. Previous studies have described nuclear and cytosolic functions of MacroD1. However, in this study we show that endogenous MacroD1 protein is highly enriched within mitochondria. We also show that MacroD1 is highly expressed in human and mouse skeletal muscle. Furthermore, we show that MacroD1 can efficiently remove ADP-ribose from 5′ and 3′-phosphorylated double stranded DNA adducts *in vitro*. Overall, we have shown that MacroD1 is a mitochondrial protein with promiscuous enzymatic activity that can target the ester bonds of ADP-ribosylated phosphorylated double-stranded DNA ends. These findings have exciting implications for MacroD1 and ADP-ribosylation within the regulation of mitochondrial function and DNA-damage *in vivo*.

## Introduction

ADP-ribosylation is a chemical modification and is involved in the regulation of a number of processes including stress response, transcription, chromatin structure, DNA damage repair, cell division and apoptosis (Gibson and Kraus, 2012; Barkauskaite et al., 2015; Gupte et al., 2017; Palazzo et al., 2017). ARTs are enzymes that synthesize ADP-ribosylation and, to date, all known ARTs utilize NAD^+^ as a substrate to transfer ADPr onto their molecular targets. The known ARTs can be broadly classified into several different protein families such as (1) ARTDs, also commonly known as PARPs, (2) ARTCs, bearing a related fold to PARPs, and (3) Sirtuins, a family of NAD^+^ dependent deacetylases with some members known to ADP-ribosylate proteins (He et al., 2012; Dölle et al., 2013; Choi and Mostoslavsky, 2014; Rack et al., 2015; Gupte et al., 2017; Palazzo et al., 2017).

Poly(ADP-ribose) polymerases are one of the most widely studied ART groups. In humans, there are 17 different PARPs with different sub-cellular localizations including plasma membrane, Golgi, endoplasmic reticulum, nucleus, nucleolus and cytosol (Leung, 2014; Perina et al., 2014; Vyas et al., 2014; Bock and Chang, 2016; Abplanalp and Hottiger, 2017). Mammalian PARPs have been most extensively studied within the context of DNA repair, however, they have also been implicated in chromatin remodeling, transcription, unfolded-protein response, cellular stress response, host–virus interactions and many more (Gupte et al., 2017). Some members of the PARP superfamily, such as PARP1/2 and Tankyrases (PARP5a and PARP5b), can make PAR chains while most other PARP family members have MART activity (D’Amours et al., 1999; Feijs et al., 2013; Vyas et al., 2014; Pascal and Ellenberger, 2015).

ADP-ribosylation is a reversible modification and several hydrolase enzymes have been described that can hydrolyze ADPr adducts. In mammals, ADP-ribosyl hydrolase enzymes can be broadly classified into two groups based on their catalytic domains as (1) macrodomain containing or (2) DraG-like fold containing hydrolases. Currently, macrodomain containing hydrolases are the most well characterized and are PARG, MacroD1, MacroD2 and TARG1. While these belong to the same family, the have different specificities and reaction mechanisms (Barkauskaite et al., 2015). PARG is one of the most widely studied hydrolases and has been shown to rapidly catalyze the cleavage of PAR chains by hydrolyzing the *O*-glycosidic bond between the ADPr units, but is unable to hydrolyze the terminal ADPr unit linked directly to a protein (Alvarez-Gonzalez and Althaus, 1989; Lin et al., 1997; Slade et al., 2011; Barkauskaite et al., 2013). TARG1, MacroD1, and MacroD2 hydrolyze mono-ADPr (MAR) at glutamic or aspartic acid residues and can also hydrolyze *O*AADPr, a by-product of sirtuin activity (Chen et al., 2011; Peterson et al., 2011; Jankevicius et al., 2013; Rosenthal et al., 2013; Sharifi et al., 2013; Rack et al., 2016). ARH 1–3 are mammalian homologs containing the DraG-like fold. ARH3 has been shown to hydrolyze PAR chains and *O*AADPr (Oka et al., 2006; Ono et al., 2006). Recently, ARH3 has been shown to efficiently remove ADP-ribosylation on serine residues, a modification that is catalyzed by PARP1/HPF1 and PARP2/HPF1 complexes that regulate genome stability (Gibbs-Seymour et al., 2016; Bonfiglio et al., 2017; Fontana et al., 2017). ARH1 specifically removes ADPr from arginine residues (Kato et al., 2007). Conversely, ARH2 has no known hydrolase activity. Two additional families of evolutionarily distinct hydrolases are (1) NUDIX family proteins - hNUDT16 and *Escherichia coli* RppH and (2) ENPP family proteins – ENPP1 and svPDE1 can also catalyze removal of MAR or PAR signal by hydrolysis of ADPr phosphodiester bonds (Palazzo et al., 2015, 2016; Daniels et al., 2016). To date, there are few examples of enzymatic reversal of DNA ADP-ribosylation, DarG antitoxin partner of DarT DNA ART and PARG in removal of DNA PARylation catalyzed by PARP1/2 (Jankevicius et al., 2016; Talhaoui et al., 2016). Recently it has been demonstrated that PARP3 can mono-ADP-ribosylate double-stranded DNA ends and that several cellular hydrolases, such as PARG, MACROD2, TARG1, and ARH3, can reverse this modification (Munnur and Ahel, 2017).

The crystal structure of human ADP-ribosyl hydrolase MacroD1 (residues 91–325) has been solved and consists of a macrodomain (residues 151–322) and an N-terminal region (residues 91–136) rich in basic residues (Chen et al., 2011). The macrodomain consists of a three-layered α-β-α sandwich with a central six-stranded β-sheet. Structure-based sequence alignments indicate that human MacroD1 is highly related to YmdB protein from *E. coli*, suggesting that they are functional and structural homologs (Perina et al., 2014). MacroD1 has been shown to deacetylate *O*AADPr, producing ADPr and acetate, and can also hydrolyze ADPr-1″phosphate (ADPr-phosphate) (Neuvonen and Ahola, 2009; Chen et al., 2011). Finally, MacroD1 can hydrolyze MAR but not PAR chains, from PARP1 and PARP10 automodified proteins *in vitro* (Barkauskaite et al., 2013; Jankevicius et al., 2013; Rosenthal et al., 2013). Site-directed mutagenesis analysis showed that a highly conserved glycine residue (G270 in humans) is required for both *O*AADPr deacetylase and MAR hydrolase activity (Chen et al., 2011; Barkauskaite et al., 2013). Despite this knowledge of MacroD1 biochemical activities *in vitro*, the function of MacroD1 *in vivo* is yet to be deciphered. Earlier studies have implicated MacroD1 expression (previously known as LRP16 – leukemia related protein 16) in the pathophysiology of several human cancers (Xi, 2010; Zhao et al., 2010; Shao et al., 2015). Han et al. (2003, 2007) showed that estrogen (17β-estradiol) treatment increased MacroD1 mRNA expression and cell proliferation in breast cancer MCF-7 cells via ERα activation and that MacroD1 was required for the estrogen-responsive proliferation ability of MCF-7 cells. Furthermore, it has been shown that MacroD1 physically interacts with ERα (Han et al., 2007). It has also been suggested that MacroD1 is a co-activator of AR in LNCaP prostate cancer cells, as binding of MacroD1 to AR (via the macrodomain) is required to amplify the transcriptional activity of AR upon androgen treatment (Yang et al., 2009). MacroD1 also integrates into the NF-κB transcriptional complex by associating with p65 and is required for NF-κB dependent gene expression (Wu et al., 2011).

In a previous study, overexpressed C-terminal tagged MacroD1 was shown to localize to mitochondria, however, the sub-cellular localization of endogenous MacroD1 is still unknown (Neuvonen and Ahola, 2009). In this study we aimed to determine the exact subcellular localization of MacroD1 and to gain insight into the physiological and cellular function of the endogenous MacroD1 protein. We show that MacroD1 is primarily a mitochondrial protein, located within the MM and that the N-terminal region (residues 1–77) of MacroD1 protein is required for mitochondrial localization. Furthermore, we show that MacroD1 is differentially expressed in a tissue-specific manner in human and mouse tissues and human cancer cell lines. We show that MacroD1 is highly expressed in skeletal muscle, a tissue with high mitochondrial content, consistent with a functional role of MacroD1 within mitochondria *in vivo*. Finally, we have biochemically characterized the human MacroD1 protein and, together with the previously published data, conclude that MacroD1 is a promiscuous ADPr hydrolase that removes a wide range of ADP-ribosylated adducts with ester bonds including proteins, DNAs and small chemical groups (Rack et al., 2016).

## Materials and Methods

### Immunoblotting

Mouse tissue and human cell protein extractions were performed using RIPA buffer [150 mM NaCl, 1% Triton X-100, 0.5% sodium deoxycholate, 0.1% SDS, 50 mM Tris-HCl (pH 8.0)] supplemented with protease inhibitors (Roche) and phosphatase inhibitors (Roche). Snap frozen mouse tissues were homogenized using a MP FastPrep-24 homogeniser (MP Biomedicals). To ensure equal loading, protein lysate concentrations were determined using Bio-Rad protein assay dye reagent concentrate (Bio-Rad) according to the manufacturer’s instructions. Protein samples were diluted to equal concentrations before addition of 4 X NuPage LDS sample buffer (Thermo Fisher) and heat denatured at 98°C for 5 min. Equal volumes of samples were loaded onto precast 4–12% Bis-Tris NuPage Polyacrylamide gels (Thermo Fisher). Electrophoresis was performed for 50 min at 180 V using the XCell Surelock Mini Cell tank system (Thermo Fisher) and MOPS running buffer (Thermo Fisher). Proteins were transferred onto nitrocellulose membranes using Trans-Blot Turbo Transfer System (Bio-Rad). Commercially available primary antibodies used: HSP60 (Santa Cruz), DNA ligase III (Novus Bio), GAPDH (Millipore), ATP5A (Abcam), MACROD1 (Abcam) and β-tubulin (Abcam). Blocking with 5% Milk PBST for 1 h at RT, primary antibodies were diluted in 5% Milk-PBST and incubated overnight at 4°C, secondary antibodies were diluted in 5% Milk PBST for 1 h at RT.

### Plasmids

Human full length MacroD1 was amplified from a human HeLa cDNA library and cloned into pDONR221 (Thermo Fisher) entry vector and a N-terminal truncation mutant was generated the same way by excluding the first 77 amino acids using a different N-terminal primer. SCO6450 (UniProt: Q9ZBG3) was cloned from total *Streptomyces coelicolor* DNA. For transient transfection in human cells, full-length and truncated MacroD1 pDONR221 vectors were recombined using the Gateway LR reaction (Thermo Fisher) into the pDEST47 destination vector for the expression of C-terminal GFP fused proteins in human cell lines. PARP1 EQ was expressed in pET28 vector and was purified as previously described (Sharifi et al., 2013). DarT was expressed in pBAD vector, transformed into BL21 strains, induced with arabinose and purified using TALON affinity resin (Clontech) as previously described (Jankevicius et al., 2016). Macrodomain proteins were expressed in pDEST17 or pET15b and purified as previously described (Chen et al., 2011).

### Human Cell Culture and Imaging

All human cell lines used in this study were cultured in DMEM (Sigma) supplemented with 10% FBS (Thermo Fisher) and penicillin/streptomycin (Thermo Fisher) at 37°C with 5% CO_2_. Transient DNA transfections (MacroD1-GFP and Δ77MacroD1-GFP) were performed using TransIT-LT1 Transfection Reagent (Mirus Bio) and transient small interfering RNA (siRNA) transfections (control siRNA, MacroD1 siRNA 1 and MacroD1 siRNA 2) were performed with Lipofectamine RNAiMAX (Thermo Fisher) each according to the manufacturer’s instructions. For endogenous imaging of MacroD1, RD cells were seeded on coverslips in 24-well plates and treated with control siRNA, MacroD1 siRNA 1 or MacroD1 siRNA 2 for 96 h using Lipofectamine RNAiMAX (Thermo Fisher). Cells were washed 4 times in PBS and fixed in 4% formalin (Sigma). Cell coverslips were permeabilized using PBS supplemented with 0.5% Triton X-100 (Sigma) for 5 min and blocked with PBS supplemented with 10% goat serum (Sigma) for 1 h before primary and secondary antibodies were applied each for 1 h. Nuclei were counterstained with DAPI (Thermo Fisher) for 5 min before imaging. Primary antibodies: ATP5A (Abcam, ab14748) and MACROD1 (Abcam, ab122688); secondary antibodies: Alexa Fluor 488 goat anti-rabbit and Alexa Fluor 594 goat anti-mouse (both Thermo Fisher). For MacroD1-GFP and Δ77MacroD1-GFP localization experiments, U2OS cells were plated in glass-bottomed 24-well plates before being transfected with MacroD1-GFP or Δ77MacroD1-GFP expression vectors using TransIT-LT1 Transfection Reagent (Mirus Bio) according to the manufacturer’s instructions for 48 h. Transfected U2OS cells were then incubated with complete media supplemented with 100 nM MitoTracker Deep Red FM (Thermo Fisher) for 15 min at 37°C with 5% CO_2_ to label mitochondria. Cell nuclei were counterstained with 1 μg/mL Hoechst 33258 (Thermo Fisher) diluted in PBS for 30 min at 37°C with 5% CO_2_. Following mitochondrial and nuclear labeling, live cell imaging was performed at 37°C with 5% CO_2_. Images of live and fixed cells were taken on the Olympus Fluoview FV1000 confocal microscope using a 100× oil objective.

### Mitochondrial Isolations and Proteinase K Digest

High purity mitochondrial preparations were isolated from HeLa CC cells using the Qproteome Mitochondrial Isolation Kit (Qiagen) according to the manufacturer’s instructions. The PK digest assay was adapted from a protocol previously described (Aerts et al., 2015). Briefly, HeLa cells were harvested from 15 cm^2^ dishes and resuspended in isolation Buffer (0.6 M mannitol, 10 mM Tris pH 7.4 and 1 mM EGTA). Cell homogenization was completed using a glass Teflon Dounce homogenizer. Cell homogenates were centrifuged at 400 *g* for 10 min to pellet cell debris and unbroken cells. The supernatant was then centrifuged at 11,000 *g* to pellet the mitochondria and were washed twice with isolation buffer. A Bradford Assay was performed to determine mitochondrial protein concentrations. Isolated mitochondria were pelleted again by centrifugation at 11,000 *g* and washed twice with either isolation buffer or hypotonic buffer (2 mM HEPES, pH 7.4). Triton X-100 was added to a final concentration of 1% before addition of PK (100 μg/ml) as indicated. All samples were then incubated on an end-over-end shaker for 15 min at 4°C. 5 mM PMSF was added to all samples to inhibit PK. NuPage LDS sample buffer (Thermo Fisher) was added to each sample before heat denaturation at 98°C for 5 min. Immunoblotting was performed as described above.

### MacroD1 Mouse Model

The Macrod1 KO mouse strain was a KOMP-Regeneron (Velocigene) definitive null design (project ID: VG13617) whereby exons 1–3 are deleted and replaced with the promoter driven Zen_Ub1 cassette (flanked by *LoxP* sites). Mice were generated by blastocyst injection using a pre targeted ES cell line ordered from the IMPC (International Mouse Phenotyping Consortium) (Dickinson et al., 2016). Prior to further analysis the promoter driven Zen_Ub1 cassette was removed by crossing onto a *Sox2Cre* line. All experiments were conducted under the authority of a valid United Kingdom Home Office Project License 30/3307 and have undergone due ethical review process. Tissue samples were collected from 11 to 13 weeks old, sex matched mice and using littermate controls, genetic background is C57BL/6JN mix.

### DNA ADP-Ribosylation and De-ADP-Ribosylation Assays

ADP-ribosylation of thymidine base on single stranded DNA (GTGGCGCGGAGACTTTCAGAA) by DarT was performed as previously described (Jankevicius et al., 2016). ADP-ribosylation of phosphorylated double stranded DNA ends was adapted from an earlier study (Talhaoui et al., 2016). Briefly, double-stranded DNA substrate was prepared by annealing phosphorylated or non-phosphorylated 21mer oligo (GTGGCGCGGAGACTTAGAGAA) with annealing partner 40mer (GGAATTCCCCGCGCCAAATTTCTCTAAGTCTCCGCGCCAC) at 98°C for 5 min and gradually cooled down to room temperature. Single-stranded and double-stranded DNA substrates were ADP-ribosylated in the presence of 1 μM DarT or PARP1 E998Q, respectively, and 20 mM HEPES-KOH (pH 7.6), 50 mM KCl, 5 mM MgCl_2_, 1 mM DTT, and 100 μg/ml BSA, 50 μM NAD (Trevigen) and 50 kBq ^32^P labeled NAD (PerkinElmer) per reaction. Modified DNA substrate was treated with PK and SDS or PARPi (Olaparib) and was used as a substrate for de-ADP-ribosylation reactions by adding 1 μM hydrolase enzymes (MacroD1 WT or MacroD1 mutant or SCO6450 or DarG). DNA ADP-ribosylation was performed at 37°C for 30 min and de-ADP-ribosylation assay was performed at 30°C for 30 min. The samples were loaded on a pre-run denaturing urea PAGE gel at 10–12 W in 0.5 × TBE buffer. The gel was dried under vacuum and visualized by autoradiography.

## Results

### MacroD1 Is Highly Expressed in Human and Mouse Skeletal Muscle

**FIGURE 1:**
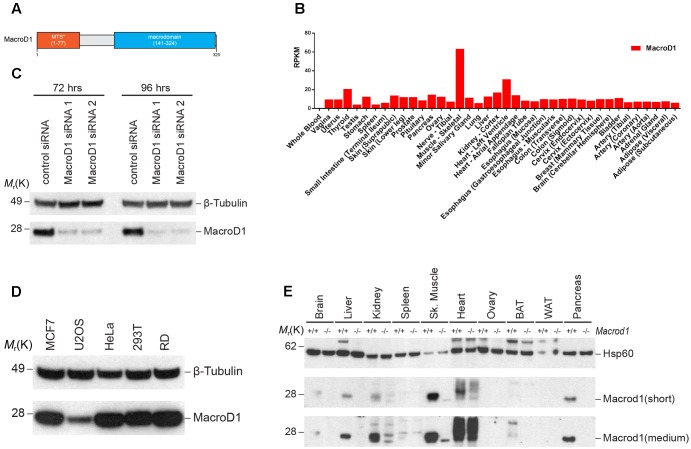
MacroD1 is differentially expressed in human and mouse. **(A)** Schematic diagram of human MacroD1 macrodomain. MTS^∗^ – predicted mitochondrial targeting sequence using MitoProt II – v1.101 (Claros and Vincens, 1996). **(B)** RNA-seq analysis of *MacroD1* (red) mRNA transcript levels in human tissues. Data from GTEx (http://www.gtexportal.org). Data shown as median mRNA transcript levels. RPMK – reads per kilobase per million mapped reads. **(C)** Immunoblot analysis of MacroD1 steady state protein levels in HeLa cells following 72 or 96 h *MacroD1* gene silencing by control siRNA, MacroD1 siRNA 1 or MacroD1 siRNA 2. **(D)** Immunoblot analysis of MacroD1 steady state protein levels in human cell lines. **(E)** Immunoblot analysis of Macrod1 steady state protein levels in tissues from *Macrod1*^+/+^ and *Macrod1*^-/-^ mice. BAT, brown adipose tissue; WAT, white adipose tissue; short, short exposure time; medium, medium exposure time.

It is not clear how MacroD1 functions in cells. The secondary and tertiary structures of MacroD1 do not unveil much information about this protein. The only known domains are the macrodomain and a predicted MTS at the N-terminus (**Figure 1A**). Whilst there is some evidence that MacroD1 expression regulates cell proliferation in human breast cancer MCF-7 cells, little is known about the physiological role of MacroD1 in humans. In order to gain insight into the possible physiological role of MacroD1, we investigated the pattern of expression in human and mouse tissues. RNA-seq data from the Genotype-Tissue Expression (GTEx) project^1^ showed that MacroD1 was highly expressed in human skeletal muscle relative to other tissues suggestive of a tissue-specific function of MacroD1 in skeletal muscle *in vivo* (**Figure 1B**). In order to validate this observation, we analyzed MacroD1 protein expression in human cell lines and mouse tissues via western blot analysis. First, we verified the specificity of the antibody using two different MacroD1 siRNAs (**Figure 1C**). We then generated a panel of protein lysates from commonly used human cell lines to determine whether MacroD1 protein expression is cell line-specific (**Figure 1D**). MacroD1 was expressed in all human cell lines used to varying levels. MacroD1 was highly overexpressed in the RD cell line, relative to osteosarcoma (U2OS) cells where only low MacroD1 protein expression was detected. High MacroD1 protein levels in RD cells are further evidence of a possible (patho)physiological role of MacroD1 in human skeletal muscle. These data indicate that MacroD1 is differentially regulated and expressed in different human cell lines and may, therefore, have cell-type specific functions. Given the marked tissue-specific pattern of MacroD1 mRNA expression in humans, we sought to determine whether this was conserved across species and at a protein level. To do this we extracted protein from wild-type (*Macrod1*^+/+^) and knock-out (*Macrod1*^-/-^) mice tissues and performed immunoblot analysis. Consistent with human mRNA expression data, Macrod1 steady state protein levels varied greatly in mouse tissues (**Figure 1E**). Macrod1 protein was highly expressed in skeletal muscle consistent with human mRNA expression data and high protein levels in the human RD cells. Macrod1 was also expressed in other tissues from *Macrod1*^+/+^ mice such as liver and pancreas, albeit at substantially lower levels (**Figure 1E**). Conversely we did not detect a MacroD1 band in white adipose tissue (WAT) or ovarian tissue from *Macrod1*^+^/^+^ mice. Additional bands were detected in both *Macrod1*^+/+^ and *Macrod1*^-/-^ mouse kidney, spleen and skeletal muscle samples due to non-specific antibody binding. In *Macrod1*^+/+^ BAT, only a faint band Macrod1 band at 28 kDa was observed. However, an additional band was observed at a higher molecular weight in *Macrod1*^+^/^+^ BAT but not in *Macrod1*^-^/^-^ BAT. There are several possible explanations for the additional band in *MacroD1*^+/+^ BAT. For example, Macrod1 may be post-translationally modified in BAT that could influence gel migration. Alternatively, it is possible that the higher molecular weight band corresponds to full-length, unprocessed MacroD1 that has retained its MTS. Furthermore, there may be a splice variant of Macrod1 in BAT that encodes an additional Macrod1 isoform with a greater molecular weight. Further experiments would be required to clarify the identity of this additional band and are beyond the scope of this study. Overall, these data suggest that the pattern of MacroD1 expression in human and mouse tissues are conserved, and indicate that MacroD1 likely has a tissue-specific function in skeletal muscles *in vivo*.

### Endogenous MacroD1 Is Highly Enriched in Mitochondria

**FIGURE 2:**
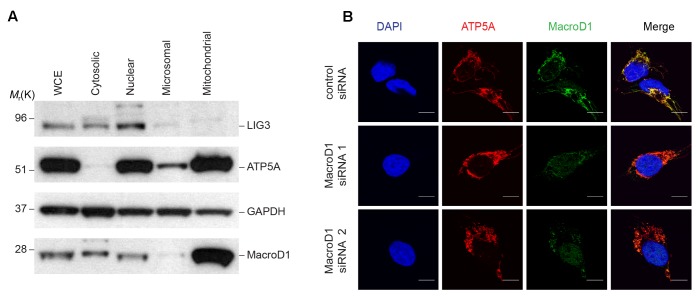
Endogenous MacroD1 localizes to mitochondria. **(A)** Immunoblot analysis of endogenous MacroD1 protein in whole cell extracts (WCE) and cytosolic [glyceraldehyde 3-phosphate dehydrogenase (GAPDH)], nuclear [DNA ligase 3 (LIG3)], microsomal and mitochondrial [ATP synthase, H+ transporting, mitochondrial F1 complex, alpha subunit 1, cardiac muscle (ATP5A)] sub-cellular fractions from HeLa cells. **(B)** Indirect immunofluorescence imaging of RD cells following 96 h *MacroD1* gene silencing by control siRNA, MacroD1 siRNA 1 or MacroD1 siRNA 2 co-stained with MacroD1 (green), ATP5A (red), and DAPI (blue). Scale bar = 10 μm.

Previous studies addressing MacroD1 function have utilized MacroD1 overexpression constructs and have documented, somewhat, opposing conclusions. Several studies have demonstrated that MacroD1 binds to and regulates several hormone receptors known to reside within the cytosol and nucleus. Conversely, another study used a C-terminal tagged MacroD1 overexpression construct and showed MacroD1 localized to mitochondria (Neuvonen and Ahola, 2009). Therefore, we sought to determine the subcellular localization of endogenous MacroD1 in order to gain insight into its possible physiologically relevant function(s). To do this we analyzed MacroD1 levels in subcellular fractions from HeLa cells (**Figure 2A**). Endogenous MacroD1 was highly enriched in mitochondrial fractions relative to whole cell lysates, similarly to the mitochondrial marker ATP5A, showing that endogenous MacroD1 is highly enriched in mitochondria (**Figure 2A**). By comparison MacroD1 steady state protein levels in cytosolic and nuclear fractions were either comparable to or less than whole cell extracts (**Figure 2A**). In order to validate this result and confirm that endogenous MacroD1 localizes to mitochondria *in situ*, we performed indirect immunofluorescence imaging in RD cells co-stained with MacroD1 and ATP5A1 antibodies (**Figure 2B**). To ensure that the MacroD1 antibody used was specific to native MacroD1, MacroD1 knock-down was performed using two MacroD1 siRNAs. MacroD1 fluorescence signal was greatly reduced in RD cells treated with either MacroD1 siRNA 1 or MacroD1 siRNA 2 relative to control siRNA showing that the antibody used was specific to MacroD1 (**Figure 2B**). Furthermore, MacroD1 fluorescence signal highly co-localized with the mitochondrial marker ATP5A1 (red) in RD cells treated with siRNA control (**Figure 2B**). A weak MacroD1 fluorescent signal was observed within the nucleus of RD cells treated with control siRNA, MacroD1 siRNA 1 and MacroD1 siRNA 2. There was no observable difference in the intensity of the nuclear signal following MacroD1 siRNA knock-down suggestive of non-specific low level background antibody labeling. However, it is also possible that a small pool of MacroD1 is located within the nucleus and has a longer cellular half-life than the mitochondrial pool of MacroD1, which could also explain the nuclear signal observed. Nevertheless, this data clearly showed that endogenous MacroD1 localizes to mitochondria *in situ*.

**FIGURE 3:**
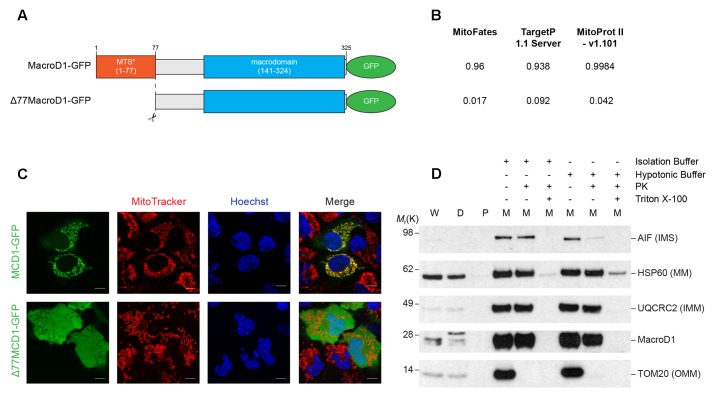
The N-terminus of MacroD1 is required for mitochondrial localization and import. **(A)** Schematic diagram of full-length MacroD1 (MacroD1-GFP) and N-terminal truncated MacroD1 (Δ77MacroD1-GFP) constructs. MTS^∗^ – Predicted mitochondrial targeting sequence was ascribed using the MTS cleavage site as predicted by MitoFates, located between amino acid 77 and 78 (Fukasawa et al., 2015). **(B)** MTS probability prediction scores for MacroD1-GFP and Δ77MacroD1-GFP cDNA sequences using MitoFates (Fukasawa et al., 2015), TargetP 1.1 Server (Emanuelsson et al., 2000) and MitoProt II – v1.101 (Claros and Vincens, 1996). **(C)** Live cell imaging of U2OS cells transfected with MacroD1-GFP (green) or Δ77MacroD1-GFP (green) for 48 h; mitochondria and nuclei were labeled with MitoTracker Deep Red FM (red) and Hoechst 33258 (blue), respectively. **(D)** Isolated mitochondrial fractions from HeLa cells were resuspended in isolation buffer or hypotonic buffer (2 mM HEPES) before treatment with proteinase K (PK) and/or Triton X-100 (as indicated). Immunoblot analysis was performed to determine PK accessibility to MacroD1 and sub-mitochondrial protein markers: AIF [apoptosis-inducing factor 1, mitochondrial; intermembrane space (IMS) marker, HSP60 (60 kDa heat shock protein, mitochondrial; mitochondrial matrix (MM) marker], UQCRC2 [cytochrome b-c1 complex subunit 2, mitochondrial; inner mitochondrial membrane (IMM) marker] and TOM20 [mitochondrial import receptor subunit; outer mitochondrial membrane (OMM)] marker. W, whole cell extract; D, cell debris; P, post mitochondrial supernatant; M, isolated mitochondrial fraction.

The vast majority of the mitochondrial proteome is encoded by nuclear DNA (nDNA), synthesized in the cytosol and trafficked to mitochondria. The majority of nDNA encoded mitochondrial proteins have an N-terminal MTS. The MTS is required for protein trafficking to mitochondria, recognition and import by the mitochondrial translocase machinery, and sub-mitochondrial protein sorting. Mitochondrial proteins destined for the IMS, IMM, and MM are synthesized as precursor proteins and the N-terminal MTS is cleaved following import (MacKenzie and Payne, 2007). MacroD1 differs from other macrodomain proteins, such as MacroD2 and TARG1 (OARD1/C6orf130), as it has an extended N-terminal sequence. The N-terminal sequence of MacroD1 contains a predicted MTS and has previously been shown to be required for mitochondrial localization (Neuvonen and Ahola, 2009). To confirm that the N-terminus of MacroD1 is required for mitochondrial localization we generated a full-length MacroD1 overexpression construct (MacroD1-GFP) and an N-terminal MacroD1 truncation overexpression construct (Δ77MacroD1-GFP) without the coding sequence for amino acids 1–77 inclusive, both tagged at the C-terminus with GFP (**Figure 3A**). Using three different *in silico* subcellular localization prediction programs MitoFates (Fukasawa et al., 2015), TargetP 1.1 (Emanuelsson et al., 2000) and MitoProt II (Claros and Vincens, 1996), we determined whether the N-terminus of MacroD1 was predicted to be a MTS (**Figure 3B**). As expected, all three servers predicted that MacroD1-GFP contained an N-terminal MTS with high confidence (**Figure 3B**). However, truncation of the N-terminus of MacroD1, as in Δ77MacroD1-GFP, was not predicted to contain a MTS in agreement with the conclusion that the N-terminus of MacroD1 is required for mitochondrial localization (**Figure 3B**). We then transiently transfected U2OS cells with full-length and truncated MacroD1 overexpression plasmids and assessed trafficking to mitochondria (MitoTracker) (**Figure 3C**). MacroD1-GFP was highly co-localized to mitochondria, however, no mitochondrial co-localization was observed in cells transfected with the MacroD1 N-terminal truncation plasmid (**Figure 3C**). These results confirm that the N-terminal sequence of MacroD1 is required for trafficking to mitochondria, consistent with previous findings by Neuvonen and Ahola, and show that amino acids 1–77 likely contain a MTS (Neuvonen and Ahola, 2009). It is of note that, similarly to **Figure 2B**, there is faint MacroD1 nuclear signal in cells transfected with MacroD1-GFP albeit at a substantially lower intensity than the strong mitochondrial signal. Overall, based on these experiments, we cannot rule out the possibility that MacroD1 is present in the nucleus at low levels, however, we can say with confidence that MacroD1 is highly enriched within mitochondria *in situ*.

To test whether MacroD1 is imported into mitochondria or is bound to the OMM, a PK accessibility assay was performed (**Figure 3D**). Isolated mitochondria were treated with PK in isolation buffer (intact mitochondria) or hypotonic buffer (mitoplasts) conditions. Hypotonic buffer was used to disrupt the OMM by osmotic shock making proteins tethered to the outer leaflet of the IMM and IMS accessible to PK. MacroD1 was protected from PK treatment in both isolation and hypotonic buffers comparably to the IMM and MM markers UQCRC2 and HSP60, respectively (**Figure 3D**). In contrast, TOM20, located at the cytosolic side of the OMM, was degraded by PK in both isolation and hypotonic buffers and the IMS protein AIF was only susceptible to PK under hypotonic buffer conditions. This shows that isolated mitochondria were intact and treatment with hypotonic buffer made IMS proteins accessible to PK. Treatment with the detergent Triton X-100 was used to solubilize all mitochondrial membranes making all mitochondrial markers and MacroD1 susceptible to PK (**Figure 3D**). Taken together, this data demonstrates that MacroD1 is imported into mitochondria and is located within the IMM or MM.

### Activities of MACROD1 on Different ADP-Ribosylated Substrates

Having established mitochondrial localization of MacroD1, the next question was its possible physiological activity. Previous *in vitro* studies have shown several activities of MacroD1. MacroD1 can hydrolyze mono-ADP-ribosylated glutamate and aspartate residues on proteins (Barkauskaite et al., 2013; Jankevicius et al., 2013; Rosenthal et al., 2013), deacetylate *O*AADPr (Chen et al., 2011) and hydrolyze ADPr-phosphates (Neuvonen and Ahola, 2009). MacroD1 has many functions *in vitro* indicating that it may act as a promiscuous ADPr hydrolase *in vivo*, cleansing and recycling cellular ADPr adducts. In recent years, multiple studies have demonstrated that certain DNA adducts are a substrate of some ARTs. For example, the bacterial toxin DarT has previously been shown to mono-ADP-ribosylate thymidine on single stranded DNA base in a sequence-specific manner (Jankevicius et al., 2016). In humans, PARP1 and PARP2 have been shown to ADP-ribosylate phosphorylated DNA at strand break termini *in vitro* (Talhaoui et al., 2016). To further address the promiscuity of MacroD1 we tested its activity against known ADP-ribosylated DNA adducts.

**FIGURE 4:**
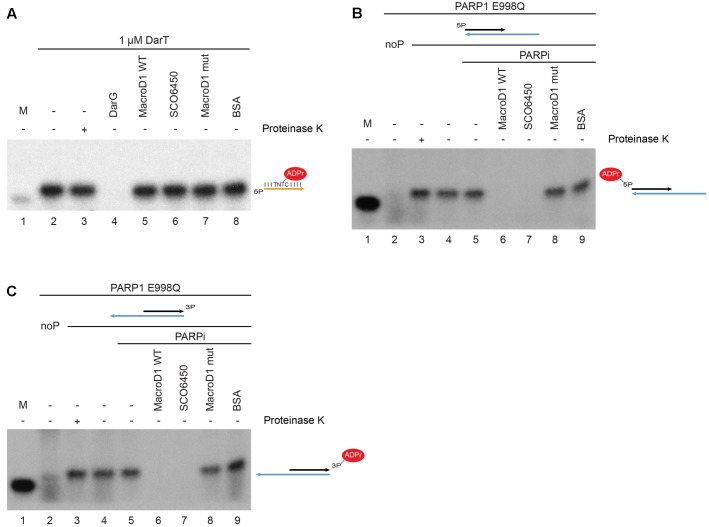
MacroD1 and *Streptomyces coelicolor* protein SCO6450 efficiently remove mono-ADP-ribose adducts from ADP-ribosylated phosphorylated double stranded DNA ends. **(A)** De-ADP-ribosylation of ADP-ribosylated thymidine base on single stranded DNA by DarG (lane 4), MacroD1 WT (lane 5), SCO6450 (lane 6), and MacroD1 mutant (MacroD1 G270E – lane 7) macrodomain-containing proteins. Thymidine base on single stranded DNA was ADP-ribosylated by DarT (lanes 2–8). In both **(B,C)** phosphorylated (lanes 3–9) double stranded DNA was mono-ADP-ribosylated by PARP1 E998Q. Removal of mono-ADP-ribosylated 5′ **(B)** and 3′ **(C)** phosphorylated double stranded DNA ends by MacroD1 WT (lane 6), SCO6450 (lane 7), and MacroD1 mutant (MacroD1 G270E – lane 8) macrodomain containing protein; ddH_2_O (lane 5) and BSA (lane 9) were used as negative controls. PARP1 E998Q was unable to ADP-ribosylate non-phosphorylated double stranded DNA (noP) (lane 2). Mono-ADP-ribosylated phosphorylated double-stranded DNA samples were treated with (lane 3) or without PK (lane 4) to confirm DNA and not protein was the substrate of PARP1 E998Q modification. ^32^P radiolabelled 21mer DNA was used as a size marker (M, lane 1 in all experiments). PARPi, PARP inhibitor; BSA, bovine serum albumin.

First we prepared ADP-ribosylated thymidine on single stranded DNA, as previously described (Jankevicius et al., 2016), and tested whether MacroD1 could act on this substrate (**Figure 4A**). The single stranded 21mer oligonucleotide used contained a single DarT modification motif (TNTC). The 21mer substrate oligonucleotide was modified with DarT and ^32^P NAD^+^ was used as a radioactively labeled ADPr donor. ^32^P-labeled 21mer oligonucleotide was used as a size marker (lane 1). As expected, the 21mer substrate oligonucleotide was efficiently modified by DarT (lane 2) even following PK treatment (lane 3) showing that DNA was the substrate modified by DarT. Consistent with the literature, the bacterial antitoxin DarG efficiently removed ADPr from the DarT modified 21mer single stranded DNA substrate (lane 4) (Jankevicius et al., 2016). Conversely, wild-type MacroD1 (MacroD1 WT), SCO6450 (a macrodomain-containing protein SCO6450 found in *S. coelicolor*, a close bacterial homolog of human MacroD1) and a mutant MacroD1 (lanes 5–7) did not remove ADPr from DarT modified single-stranded DNA. These results show that MacroD1 and SCO6450 cannot remove ADPr from ADP-ribosylated thymidine on single stranded DNA and that the reversal of this modification is likely specific to the toxin–antitoxin DarG/DarT system found in bacteria.

Next we prepared mono-ADP-ribosylated phosphorylated double stranded DNA ends, as previously described (Talhaoui et al., 2016) and tested whether MacroD1 could act on this substrate. ADP-ribosylated phosphorylated double stranded DNA ends were prepared as previously described with the following changes (Palazzo et al., 2017). We used the PARP1 E998Q (PARP1 EQ) mutant to specifically introduce MAR adducts, rather than chains of ADPr, to DNA substrates (Marsischky et al., 1995; Tao et al., 2009). ^32^P NAD^+^ was used as a radioactively labeled ADPr donor. As for the DNA substrates, we used double-stranded 5′ (**Figure 4B**) or 3′-phosphate (**Figure 4C**) 21mer oligonucleotide annealed with a longer 40mer fragment; as they were shown to be efficiently modified *in vitro* (Talhaoui et al., 2016; Palazzo et al., 2017). As seen in **Figures 4B,C**, PARP1 EQ only modified 5′ and 3′-phosphorylated double stranded DNA ends (lane 3) but not non-phosphorylated double stranded DNA ends (lane 2). The modified phosphorylated DNA ends remained intact following PK digest (lane 3) confirming that DNA modification rather than protein modification. Importantly, we could demonstrate that wild-type human MacroD1 (MacroD1 WT) efficiently removes MAR from both 5′ and 3′-phosphorylated-DNA (lane 6), while a catalytic-mutant form of human MacroD1 (MacroD1 G270E) cannot (lane 8) (Chen et al., 2011). Together this data shows that MacroD1 can remove mono-ADP-ribosylation from both 5′ and 3′ phosphorylated double stranded DNA, dependent upon the hydrolase activity of its macrodomain, as recently noted for several other human ADP-ribosyl hydrolases (Palazzo et al., 2017). Interestingly, SCO6450, a bacterial homolog of human MacroD1, also efficiently removed mono-ADP-ribosylation from 5′ and 3′-phosphorylated DNA substrates (lane 7). The ability of macrodomain-containing proteins from both human and bacteria to remove ADPr from phosphorylated double stranded DNA, indicates that this novel and recently described function is evolutionarily conserved and possibly of physiological relevance/importance.

## Discussion

Several studies have described nuclear and cytosolic functions of MacroD1 *in vivo*. For instance, MacroD1 has been shown to bind and regulate proteins such as the transcription factors ERα, AR and NF-κB (Han et al., 2007; Yang et al., 2009; Wu et al., 2011). More recently, MacroD1 has been shown to bind and selectively activate, the primarily cytosolic protein (Jeffrey et al., 1995), Protein Kinase R (PKR) (Li et al., 2017). In this study we have shown that endogenous MacroD1 is highly enriched in and primarily localizes to mitochondria. It is, therefore, unsurprising that MacroD1 is highly expressed in human and mouse skeletal muscle, a tissue with high mitochondrial content and function. Our data is supported by evidence from MitoCarta and MitoMiner, databases of human nDNA and mtDNA genes encoding mitochondrial proteins, both categorizing MacroD1 as a mitochondrial protein (Smith and Robinson, 2009; Calvo et al., 2016). Furthermore, several independent proteomic analyses characterizing the mitochondrial proteome reported enrichment of MacroD1 within mitochondrial fractions, further supportive of the findings presented within this paper (Lefort et al., 2009; Rhee et al., 2013). Our data, together with several others, are consistent with the premise that MacroD1 has a physiological function within mitochondria *in vivo*.

Furthermore, we confirm that the N-terminus of overexpressed MacroD1 is required for mitochondrial localization, in agreement with previous findings (Neuvonen and Ahola, 2009). The predicted size of full-length MacroD1 (1–325) is 35.5 kDa. Through the use of two different siRNAs we validated the specificity of our MacroD1 antibody and have shown that there is 1 primary isoform of MacroD1 that is approximately 28 kDa in size in all cell lines used. The difference between the observed and predicted size of MacroD1 is likely due to cleavage of the N-terminus MTS following mitochondrial import. MitoProt II predicts that there is an MTS cleavage site between amino acid 77 and 78 in MacroD1 (Claros and Vincens, 1996). If true, the resultant cleaved MacroD1 protein would be 250 amino acids long with a molecular weight of 27.5 kDa, consistent with this hypothesis. In some previous studies, it is not always clear whether N- or C-terminal tagged MacroD1 overexpression constructs have been used during analysis. However, if N-terminal tags were used, this would disrupt efficient mitochondrial localization and may account for some of the non-mitochondrial functions of MacroD1 documented. Having said this, it is also possible that a small proportion of cellular MacroD1 resides within the nucleus or cytosol (especially upon some stress) or that non-mitochondrial isoforms of MacroD1 may be differentially expressed in a cell line or tissue-specific manner *in vivo*, which could also account for these findings. After several repeated experiments overexpressing MacroD1-GFP in human cells we can confirm that we always observe some nuclear signal in this system. Further clarification of the sub-cellular location of previously reported binding partners of MacroD1 is required in order to establish true physiological relevance and to conclusively determine whether there is indeed a pool of MacroD1 within the nucleus or cytosol. Previous studies have focused on illuminating the association between MacroD1 dysregulation and cancer development. It would be interesting to determine whether dysregulation of MacroD1 localization underlies this association which would account for differences between the findings of this paper and those from previous studies.

It has only recently been discovered that double stranded phosphorylated DNA adducts can be ADP-ribosylated by PARP1/PARP2/PARP3 and de-ADP-ribosylated by several human macrodomain-containing hydrolases *in vitro* (Talhaoui et al., 2016; Munnur and Ahel, 2017; Palazzo et al., 2017). In this paper we show that MacroD1 can also remove MAR from phosphorylated double stranded DNA adducts. The physiological significance of DNA ADP-ribosylation is yet unknown. It is possible that ADP-ribosylation of DNA is in fact an off-target activity of PARP family proteins resulting in DNA lesions in a similar way to the formation of DNA adenylates produced during abortive DNA ligation events (Ahel et al., 2006). In both scenarios, 5′phosphorylated DNA traps a nuclear enzyme to produce nucleotide DNA adducts. In the case of DNA 5′P adenylates, these are processed by Aprataxin a DNA repair factor responsible for direct reversal of these lesions by restoring the conventional 5′P DNA end which can then be ligated in the presence of the DNA ligase (Ahel et al., 2006; Rass et al., 2007). If this is indeed true, then MacroD1 may function in the regulation of mitochondrial DNA-damage repair by removing ADPr from ADP-ribosylated DNA adducts.

To date, the only example of reversible DNA ADP-ribosylation in bacteria comes from the studies of toxin-antitoxin system (DarT/G) (Jankevicius et al., 2016). SCO6450, a bacterial homolog of MacroD1 (orthologs of which are also predicted to exist in most of the bacteria), has comparable hydrolase activity toward ADP-ribosylated DNA adducts as MacroD1, suggesting that modification of DNA by ADP-ribosylation may be a widespread type of signaling in a variety of organisms.

The question remains, whether MacroD1 performs the various described enzymatic ADPr hydrolase functions within mitochondria and if so, which enzyme with ART activity can generate ADPr adducts in mitochondria. There is currently limited evidence of ADP-ribosylation within mitochondria. This is primarily because no member of the PARP family has been shown to be reside within mitochondria. However, human Sirtuins 3–5 are known mitochondrial proteins with deacetylation activity, the product of which is *O*AADPr (Zhu et al., 2014). The mitochondrial function of MacroD1 may, therefore, be paired with protein deacetylation by sirtuins. In addition to this, SIRT4 has also been suggested to reversibly mono-ADP-ribosylate glutamate dehydrogenase protein in mitochondria (Haigis et al., 2006). Furthermore, there is also some evidence to suggest that non-enzymatic protein ADP-ribosylation occurs in mitochondria (Hilz et al., 2000).

Together with previous findings, we conclude that, MacroD1 is a promiscuous mitochondrial protein that can remove MAR from a number of ADP-ribosylated adducts with ester bonds including ADP-ribosylated phosphorylated double stranded DNA ends (Neuvonen and Ahola, 2009; Chen et al., 2011; Jankevicius et al., 2013; Rosenthal et al., 2013). These findings have exciting implications for MacroD1 and ADP-ribosylation in the regulation of mitochondria function(s).

## Ethics Statement

This study was carried out in accordance with the recommendations of ASPA [Animals (Scientific Procedures) Act 1986], with both Local Ethical Review Committee (ERC) (Sir William Dunn School of Pathology, University of Oxford) and the United Kingdom Home Office. The Protocol was approved by both Local ERC and the United Kingdom Home Office, which is also in compliance with the European Directive 2010/63/EU on the protection of animals used for scientific purposes.

## Author Contributions

TA performed the cell biology studies, analyzed the data and wrote the manuscript. KC developed the Macrod1 mouse model. DM, LP, and AM performed the biochemical experiments. IA conceived the study and analyzed the data.

## Conflict of Interest Statement

The authors declare that the research was conducted in the absence of any commercial or financial relationships that could be construed as a potential conflict of interest.

## Footnotes

1 www.gtexportal.org

## Abbreviations

ADPr: ADP-ribose
ADPr-phosphate: ADP-ribose-1″phosphate
AR: androgen receptor
ARH: ADP-ribosylhydrolase
ART: ADP-ribosyltransferase
ARTC: cholera toxin like ADP-ribosyltransferase
ARTD: diphtheria toxin like ADP-ribosyltransferases
ENPP: ectonucleotide pyrophosphate/phosphodiesterase
ERα: estrogen receptor alpha
IMM: inner mitochondrial membrane
IMS: intermembrane space
MAR: mono-ADP-ribose
MART: mono-ADP-ribosyltransferase
MM: mitochondrial matrix
MTS: mitochondrial targeting sequence
NAD^+^: β-nicotinamide adenine dinucleotide
NUDIX: nucleoside diphosphate linked X-moiety hydrolase
*O*AADPr: *O*-Acetyl-ADP-ribose
OMM: outer mitochondrial membrane
PAR: poly ADP-ribose
PARG: poly(ADP-ribosyl) glycohydrolase
PARPs: poly(ADP-ribose) polymerases
PK: proteinase K
RD: rhabdomyosarcoma
svPDE1: snake venom phosphodiesterase
TARG1: terminal ADP-ribosyl glycohydrolase 1

## References

[B1] Abplanalp J. Hottiger M. O. (2017). Cell fate regulation by chromatin ADP-ribosylation. *Semin. Cell Dev. Biol.* 63 114–122. 10.1016/j.semcdb.2016.09.010 27693398

[B2] Aerts L. Craessaerts K. De Strooper B. Morais V. A. (2015). PINK1 kinase catalytic activity is regulated by phosphorylation on serines 228 and 402. *J. Biol. Chem.* 290 2798–2811. 10.1074/jbc.M114.620906 25527497 PMC4317039

[B3] Ahel I. Rass U. El-Khamisy S. F. Katyal S. Clements P. M. McKinnon P. J. (2006). The neurodegenerative disease protein aprataxin resolves abortive DNA ligation intermediates. *Nature* 443 713–716. 10.1038/nature05164 16964241

[B4] Alvarez-Gonzalez R. Althaus F. R. (1989). Poly(ADP-ribose) catabolism in mammalian cells exposed to DNA-damaging agents. *Mutat. Res.* 218 67–74. 10.1016/0921-8777(89)90012-8 2770765

[B5] Barkauskaite E. Brassington A. Tan E. S. Warwicker J. Dunstan M. S. Banos B. (2013). Visualization of poly(ADP-ribose) bound to PARG reveals inherent balance between exo- and endo-glycohydrolase activities. *Nat. Commun.* 4:2164. 10.1038/ncomms3164 23917065 PMC3741636

[B6] Barkauskaite E. Jankevicius G. Ahel I. (2015). Structures and mechanisms of enzymes employed in the synthesis and degradation of PARP-dependent protein ADP-ribosylation. *Mol. Cell* 58 935–946. 10.1016/j.molcel.2015.05.007 26091342

[B7] Bock F. J. Chang P. (2016). New directions in poly(ADP-ribose) polymerase biology. *FEBS J.* 283 4017–4031. 10.1111/febs.13737 27087568

[B8] Bonfiglio J. J. Fontana P. Zhang Q. Colby T. Gibbs-Seymour I. Atanassov I. (2017). Serine ADP-ribosylation depends on HPF1. *Mol. Cell* 65 932.e6–940.e6. 10.1016/j.molcel.2017.01.003 28190768 PMC5344681

[B9] Calvo S. E. Clauser K. R. Mootha V. K. (2016). MitoCarta2.0: an updated inventory of mammalian mitochondrial proteins. *Nucleic Acids Res.* 44 D1251–D1257. 10.1093/nar/gkv1003 26450961 PMC4702768

[B10] Chen D. Vollmar M. Rossi M. N. Phillips C. Kraehenbuehl R. Slade D. (2011). Identification of macrodomain proteins as novel *O*-acetyl-ADP-ribose deacetylases. *J. Biol. Chem.* 286 13261–13271. 10.1074/jbc.M110.206771 21257746 PMC3075673

[B11] Choi J. E. Mostoslavsky R. (2014). Sirtuins, metabolism, and DNA repair. *Curr. Opin. Genet. Dev.* 26 24–32. 10.1016/j.gde.2014.05.005 25005742 PMC4254145

[B12] Claros M. G. Vincens P. (1996). Computational method to predict mitochondrially imported proteins and their targeting sequences. *Eur. J. Biochem.* 241 779–786. 10.1111/j.1432-1033.1996.00779.x 8944766

[B13] D’Amours D. Desnoyers S. D’Silva I. Poirier G. G. (1999). Poly(ADP-ribosyl)ation reactions in the regulation of nuclear functions. *Biochem. J.* 342 249–268. 10.1042/0264-6021:3420249 10455009 PMC1220459

[B14] Daniels C. M. Thirawatananond P. Ong S.-E. Gabelli S. B. Leungc A. K. L. (2016). Nudix hydrolases degrade protein-conjugated ADP-ribose. *Sci. Rep.* 5:18271. 10.1038/srep18271 26669448 PMC4680915

[B15] Dickinson M. E. Flenniken A. M. Ji X. Teboul L. Wong M. D. White J. K. (2016). High-throughput discovery of novel developmental phenotypes. *Nature* 537 508–514. 10.1038/nature19356 27626380 PMC5295821

[B16] Dölle C. Rack J. G. M. Ziegler M. (2013). NAD and ADP-ribose metabolism in mitochondria. *FEBS J.* 280 3530–3541. 10.1111/febs.12304 23617329

[B17] Emanuelsson O. Nielsen H. Brunak S. von Heijne G. (2000). Predicting subcellular localization of proteins based on their N-terminal amino acid sequence. *J. Mol. Biol.* 300 1005–1016. 10.1006/jmbi.2000.3903 10891285

[B18] Feijs K. L. H. Verheugd P. Lüscher B. (2013). Expanding functions of intracellular resident mono-ADP-ribosylation in cell physiology. *FEBS J.* 280 3519–3529. 10.1111/febs.12315 23639026

[B19] Fontana P. Bonfiglio J. J. Palazzo L. Bartlett E. Matic I. Ahel I. (2017). Serine ADP-ribosylation reversal by the hydrolase ARH3. *eLife* 6:e28533. 10.7554/eLife.28533 28650317 PMC5552275

[B20] Fukasawa Y. Tsuji J. Fu S. C. Tomii K. Horton P. Imai K. (2015). MitoFates: improved prediction of mitochondrial targeting sequences and their cleavage sites. *Mol. Cell. Proteomics* 14 1113–1126. 10.1074/mcp.M114.043083 25670805 PMC4390256

[B21] Gibbs-Seymour I. Fontana P. Rack J. G. M. Ahel I. (2016). HPF1/C4orf27 Is a PARP-1-interacting protein that regulates PARP-1 ADP-ribosylation activity. *Mol. Cell* 62 432–442. 10.1016/j.molcel.2016.03.008 27067600 PMC4858568

[B22] Gibson B. A. Kraus W. L. (2012). New insights into the molecular and cellular functions of poly(ADP-ribose) and PARPs. *Nat. Rev. Mol. Cell Biol.* 13 411–424. 10.1038/nrm3376 22713970

[B23] Gupte R. Liu Z. Kraus W. L. (2017). Parps and adp-ribosylation: recent advances linking molecular functions to biological outcomes. *Genes Dev.* 31 101–126. 10.1101/gad.291518.116 28202539 PMC5322727

[B24] Haigis M. C. Mostoslavsky R. Haigis K. M. Fahie K. Christodoulou D. C. Murphy A. J. (2006). SIRT4 inhibits glutamate dehydrogenase and opposes the effects of calorie restriction in pancreatic β cells. *Cell* 126 941–954. 10.1016/j.cell.2006.06.057 16959573

[B25] Han W.-D. Mu Y.-M. Lu X.-C. Xu Z.-M. Li X.-J. Yu L. (2003). Up-regulation of LRP16 mRNA by 17beta-estradiol through activation of estrogen receptor alpha (ERalpha), but not ERbeta, and promotion of human breast cancer MCF-7 cell proliferation: a preliminary report. *Endocr. Relat. Cancer* 10 217–224. 10.1677/erc.0.0100217 12790785

[B26] Han W. D. Zhao Y. L. Meng Y. G. Zang L. Wu Z. Q. Li Q. (2007). Estrogenically regulated LRP16 interacts with estrogen receptorα and enhances the receptor’s transcriptional activity. *Endocr. Relat. Cancer* 14 741–753. 10.1677/ERC-06-0082 17914104

[B27] He W. Newman J. C. Wang M. Z. Ho L. Verdin E. (2012). Mitochondrial sirtuins: regulators of protein acylation and metabolism. *Trends Endocrinol. Metab.* 23 467–476. 10.1016/j.tem.2012.07.004 22902903

[B28] Hilz H. Koch R. Fanick W. Klapproth K. Adamietz P. (2000). Nonenzymic ADP-ribosylation of specific mitochondrial polypeptides. *Proc. Natl. Acad. Sci. U.S.A.* 81 3929–3933. 10.1073/pnas.81.13.3929 6588374 PMC345341

[B29] Jankevicius G. Ariza A. Ahel M. Ahel I. (2016). The toxin-antitoxin system DarTG catalyzes reversible ADP-ribosylation of DNA. *Mol. Cell* 64 1109–1116. 10.1016/j.molcel.2016.11.014 27939941 PMC5179494

[B30] Jankevicius G. Hassler M. Golia B. Rybin V. Zacharias M. Timinszky G. (2013). A family of macrodomain proteins reverses cellular mono-ADP-ribosylation. *Nat. Struct. Mol. Biol.* 20 508–514. 10.1038/nsmb.2523 23474712 PMC7097781

[B31] Jeffrey I. W. Kadereit S. Meurs E. F. Metzger T. Bachmann M. Schwemmle M. (1995). Nuclear localization of the interferon-inducible protein kinase PKR in human cells and transfected mouse cells. *Exp. Cell Res.* 218 17–27. 10.1006/excr.1995.1126 7737357

[B32] Kato J. Zhu J. Liu C. Moss J. (2007). Enhanced sensitivity to cholera toxin in ADP-ribosylarginine hydrolase-deficient mice. *Mol. Cell. Biol.* 27 5534–5543. 10.1128/MCB.00302-07 17526733 PMC1952103

[B33] Lefort N. Yi Z. Bowen B. Glancy B. De Filippis E. A. Mapes R. (2009). Proteome profile of functional mitochondria from human skeletal muscle using one-dimensional gel electrophoresis and HPLC-ESI-MS/MS. *J. Proteomics* 72 1046–1060. 10.1016/j.jprot.2009.06.011 19567276 PMC2774790

[B34] Leung A. K. L. (2014). Poly(ADP-ribose): an organizer of cellular architecture. *J. Cell Biol.* 205 613–619. 10.1083/jcb.201402114 24914234 PMC4050725

[B35] Li X. Wu Z. An X. Mei Q. Bai M. Hanski L. (2017). Blockade of the LRP16-PKR-NF-κB signaling axis sensitizes colorectal carcinoma cells to DNA-damaging cytotoxic therapy. *eLife* 6:e27301. 10.7554/eLife.27301 28820388 PMC5562444

[B36] Lin W. Amé J. C. Aboul-Ela N. Jacobson E. L. Jacobson M. K. (1997). Isolation and characterization of the cDNA encoding bovine poly(ADP-ribose) glycohydrolase. *J. Biol. Chem.* 272 11895–11901. 10.1074/jbc.272.18.11895 9115250

[B37] MacKenzie J. A. Payne R. M. (2007). Mitochondrial protein import and human health and disease. *Biochim. Biophys. Acta* 1772 509–523. 10.1016/j.bbadis.2006.12.002 17300922 PMC2702852

[B38] Marsischky G. T. Wilson B. A. Collier R. J. (1995). Role of glutamic acid 988 of human poly-ADP-ribose polymerase in polymer formation: Evidence for active site similarities to the ADP-ribosylating toxins. *J. Biol. Chem.* 270 3247–3254. 10.1074/jbc.270.7.3247 7852410

[B39] Munnur D. Ahel I. (2017). Reversible mono-ADP-ribosylation of DNA breaks. *FEBS J.* 284 4002–4016. 10.1111/febs.14297 29054115 PMC5725667

[B40] Neuvonen M. Ahola T. (2009). Differential activities of cellular and viral macro domain proteins in binding of ADP-ribose metabolites. *J. Mol. Biol.* 385 212–225. 10.1016/j.jmb.2008.10.045 18983849 PMC7094737

[B41] Oka S. Kato J. Moss J. (2006). Identification and characterization of a mammalian 39-kDa poly(ADP-ribose) glycohydrolase. *J. Biol. Chem.* 281 705–713. 10.1074/jbc.M510290200 16278211

[B42] Ono T. Kasamatsu A. Oka S. Moss J. (2006). The 39-kDa poly(ADP-ribose) glycohydrolase ARH3 hydrolyzes O-acetyl-ADP-ribose, a product of the Sir2 family of acetyl-histone deacetylases. *Proc. Natl. Acad. Sci. U.S.A.* 103 16687–16691. 10.1073/pnas.0607911103 17075046 PMC1636516

[B43] Palazzo L. Daniels C. M. Nettleship J. E. Rahman N. McPherson R. L. Ong S. E. (2016). ENPP1 processes protein ADP-ribosylation *in vitro*. *FEBS J.* 283 3371–3388. 10.1111/febs.13811 27406238 PMC5030157

[B44] Palazzo L. Mikoč A. Ahel I. (2017). ADP-ribosylation: new facets of an ancient modification. *FEBS J.* 284 2932–2946. 10.1111/febs.14078 28383827 PMC7163968

[B45] Palazzo L. Thomas B. Jemth A. S. Colby T. Leidecker O. Feijs K. L. (2015). Processing of protein ADP-ribosylation by Nudix hydrolases. *Biochem. J.* 468 293–301. 10.1042/BJ20141554 25789582 PMC6057610

[B46] Pascal J. M. Ellenberger T. (2015). The rise and fall of poly(ADP-ribose): an enzymatic perspective. *DNA Repair* 32 10–16. 10.1016/j.dnarep.2015.04.008 25963443 PMC4522361

[B47] Perina D. Mikoč A. Ahel J. Ćetković H. Žaja R. Ahel I. (2014). Distribution of protein poly(ADP-ribosyl)ation systems across all domains of life. *DNA Repair* 23 4–16. 10.1016/j.dnarep.2014.05.003 24865146 PMC4245714

[B48] Peterson F. C. Chen D. Lytle B. L. Rossi M. N. Ahel I. Denu J. M. (2011). Orphan macrodomain protein (Human C6orf130) is an *O*-acyl-ADP-ribose deacylase: Solution structure and catalytic properties. *J. Biol. Chem.* 286 35955–35965. 10.1074/jbc.M111.276238 21849506 PMC3195580

[B49] Rack J. G. Morra R. Barkauskaite E. Kraehenbuehl R. Ariza A. Qu Y. (2015). Identification of a class of protein ADP-ribosylating sirtuins in microbial pathogens. *Mol. Cell* 59 309–320. 10.1016/j.molcel.2015.06.013 26166706 PMC4518038

[B50] Rack J. G. M. Perina D. Ahel I. (2016). Macrodomains: structure, function, evolution, and catalytic activities. *Annu. Rev. Biochem.* 85 431–454. 10.1146/annurev-biochem-060815-014935 26844395

[B51] Rass U. Ahel I. West S. C. (2007). Actions of aprataxin in multiple DNA repair pathways. *J. Biol. Chem.* 282 9469–9474. 10.1074/jbc.M611489200 17276982

[B52] Rhee H. W. Zou P. Udeshi N. D. Martell J. D. Mootha V. K. Carr S. A. (2013). Proteomic mapping of mitochondria in living cells via spatially restricted enzymatic tagging. *Science* 339 1328–1331. 10.1126/science.1230593.Proteomic 23371551 PMC3916822

[B53] Rosenthal F. Feijs K. L. Frugier E. Bonalli M. Forst A. H. Imhof R. (2013). Macrodomain-containing proteins are new mono-ADP-ribosylhydrolases. *Nat. Struct. Mol. Biol.* 20 502–507. 10.1038/nsmb.2521 23474714

[B54] Shao Y. Li X. Lu Y. Liu L. Zhao P. (2015). Aberrant LRP16 protein expression in primary neuroendocrine lung tumors. *Int. J. Clin. Exp. Pathol.* 8 6560–6565. 26261536 PMC4525870

[B55] Sharifi R. Morra R. Appel C. D. Tallis M. Chioza B. Jankevicius G. (2013). Deficiency of terminal ADP-ribose protein glycohydrolase TARG1/C6orf130 in neurodegenerative disease. *EMBO J.* 32 1225–1237. 10.1038/emboj.2013.51 23481255 PMC3642678

[B56] Slade D. Dunstan M. S. Barkauskaite E. Weston R. Lafite P. Dixon N. (2011). The structure and catalytic mechanism of a poly(ADP-ribose) glycohydrolase. *Nature* 477 616–620. 10.1038/nature10404 21892188 PMC3184140

[B57] Smith A. C. Robinson A. J. (2009). MitoMiner, an integrated database for the storage and analysis of mitochondrial proteomics data. *Mol. Cell. Proteomics* 8 1324–1337. 10.1074/mcp.M800373-MCP200 19208617 PMC2690483

[B58] Talhaoui I. Lebedeva N. A. Zarkovic G. Saint-Pierre C. Kutuzov M. M. Sukhanova M. V. (2016). Poly(ADP-ribose) polymerases covalently modify strand break termini in DNA fragments *in vitro*. *Nucleic Acids Res.* 44 9279–9295. 10.1093/nar/gkw675 27471034 PMC5100588

[B59] Tao Z. Gao P. Liu H. W. (2009). Identification of the ADP-ribosylation sites in the PARP-1 automodification domain: analysis and implications. *J. Am. Chem. Soc.* 131 14258–14260. 10.1021/ja906135d 19764761

[B60] Vyas S. Matic I. Uchima L. Rood J. Zaja R. Hay R. T. (2014). Family-wide analysis of poly(ADP-ribose) polymerase activity. *Nat. Commun.* 5:4426. 10.1038/ncomms5426 25043379 PMC4123609

[B61] Wu Z. Li Y. Li X. Ti D. Zhao Y. Si Y. (2011). LRP16 integrates into NF-κB transcriptional complex and is required for its functional activation. *PLOS ONE* 6:e18157. 10.1371/journal.pone.0018157 21483817 PMC3069058

[B62] Xi H.-Q. (2010). Clinicopathological significance and prognostic value of LRP16 expression in colorectal carcinoma. *World J. Gastroenterol.* 16 1644–1648. 10.3748/wjg.v16.i13.1644 20355243 PMC2848373

[B63] Yang J. Zhao Y. L. Wu Z. Q. Si Y. L. Meng Y. G. Fu X. B. (2009). The single-macro domain protein LRP16 is an essential cofactor of androgen receptor. *Endocr. Relat. Cancer* 16 139–153. 10.1677/ERC-08-0150 19022849

[B64] Zhao P. Lu Y. Han W. (2010). Clinicopathological significance and prognostic value of leukemia-related protein 16 expression in invasive ductal breast carcinoma. *Cancer Sci.* 101 2262–2268. 10.1111/j.1349-7006.2010.01658.x 20649898 PMC11159915

[B65] Zhu Y. Yan Y. Principe D. R. Zou X. Vassilopoulos A. Gius D. (2014). SIRT3 and SIRT4 are mitochondrial tumor suppressor proteins that connect mitochondrial metabolism and carcinogenesis. *Cancer Metab.* 2:15. 10.1186/2049-3002-2-15 25332769 PMC4203689

